# Towards a global-scale soil climate mitigation strategy

**DOI:** 10.1038/s41467-020-18887-7

**Published:** 2020-10-27

**Authors:** W. Amelung, D. Bossio, W. de Vries, I. Kögel-Knabner, J. Lehmann, R. Amundson, R. Bol, C. Collins, R. Lal, J. Leifeld, B. Minasny, G. Pan, K. Paustian, C. Rumpel, J. Sanderman, J. W. van Groenigen, S. Mooney, B. van Wesemael, M. Wander, A. Chabbi

**Affiliations:** 1 Institute of Crop Science and Resource Conservation – Soil Science and Soil Ecology, University of Bonn, Bonn, Germany; 2 Institute of Bio-and Geosciences, Agrosphere (IBG3), Forschungszentrum Jülich GmbH, Jülich, Germany; 3 The Nature Conservancy, Arlington, VA USA; 4 Wageningen, University and Research, Environmental Research, 6700 AA Wageningen, The Netherlands; 5 Chair of Soil Science, Department of Ecology and Ecosystem Management and Institute of Advanced Study (TUM-IAS), Technische Universität München, München, Germany; 6 Soil and Crop Science, School of Integrative Plant Science Cornell University, Ithaca, NY USA; 7 Institute of Advanced Study (TUM-IAS), Technical University Munich, Garching, Germany; 8 Department of Environmental Science, Policy, and Management, University of California, Berkeley, CA 94720 USA; 9 Department of Geography and Environmental Science, University of Reading, Reading, UK; 10 Carbon Management and Sequestration Center, CFAES/SENR, The Ohio State University, Columbus, OH 43210 USA; 11 Agroscope, Climate and Agriculture Group, 8046 Zurich, Switzerland; 12 School of Life and Environmental Sciences, Sydney Institute of Agriculture, The University of Sydney, Camperdown, NSW 2006 Australia; 13 Institute of Resources, Ecosystem and Environment of Agriculture, Nanjing Agricultural University, Nanjing, 210095 China; 14 Department of Soil and Crop Sciences and Natural Resource Ecology Lab, Colorado State University, Fort Collins, CO USA; 15 CNRS, Institute for Ecology and Environmental Sciences (IEES) Paris, Paris, France; 16 Woodwell Climate Research Center, Falmouth, MA 02540 USA; 17 Soil Biology Group, Wageningen University, 6700 AA Wageningen, The Netherlands; 18 O’Neill School of Public and Environmental Affairs, Indiana University, Bloomington, IN USA; 19 Earth and Life Institute, Université catholique de Louvain, Louvain La Neuve, Belgium; 20 Natural Resources and Environmental Sciences, University of Illinois at Urbana-Champaign College of Agriculture, Consumer and Environmental Sciences, Urbana, IL USA; 21 Institut National de Recherche pour l’Agriculture, l’Alimentation et l’Environnement (INRAE) Centre de Recherche Nouvelle-Aquitaine-Poitiers, (URP3F), Lusignan, France; 22 UMR ECOSYS, Centre INRAE, Versailles-Grignon, Bâtiment EGER, Thiverval-Grignon, France

**Keywords:** Biogeochemistry, Climate sciences, Ecology, Environmental sciences

## Abstract

Sustainable soil carbon sequestration practices need to be rapidly scaled up and implemented to contribute to climate change mitigation. We highlight that the major potential for carbon sequestration is in cropland soils, especially those with large yield gaps and/or large historic soil organic carbon losses. The implementation of soil carbon sequestration measures requires a diverse set of options, each adapted to local soil conditions and management opportunities, and accounting for site-specific trade-offs. We propose the establishment of a soil information system containing localised information on soil group, degradation status, crop yield gap, and the associated carbon-sequestration potentials, as well as the provision of incentives and policies to translate management options into region- and soil-specific practices.

## Abstract (web summary)

Reducing soil degradation and improving soil management could make an important contribute to climate change mitigation. Here the authors discuss opportunities and challenges towards implementing a global climate mitigation strategy focused on carbon sequestration in agricultural soils, and propose a framework for guiding region- and soil-specific management options.

## Introduction

Over the past decade (2009–18), the net global increase in anthropogenic CO_2_ emissions, after accounting for ocean and land sinks, was 4.9 Gt C yr^−1^ ^1^. It is now widely recognized that to tackle the resulting climate change, it will be necessary to employ negative emission technologies in addition to drastically reducing fossil fuel emissions^2^. Sequestering organic carbon in soil may potentially, and in a technically feasible manner, remove between 0.79 and 1.54 Gt C yr^−1^ from the atmosphere^3^ [p. 27], recognizing the substantial potential of soils in stabilizing the climate (e.g., ref. ^4^). However, to accumulate soil organic carbon (SOC) in globally relevant quantities, the world has to develop the policies and economic incentives to tap into this potential.

Soils have recently become part of the global carbon agenda for climate-change mitigation and adaptation through the launch of three high-level initiatives. These include the “4p1000 initiative”, which was launched at COP21 by UNFCC under the framework of the Lima-Paris Action Plan (LPAP) in Paris on December 1, 2015. The name of the initiative reflects that a comparatively small proportional increase (4‰) of the global SOC stocks in the top 0.3–0.4 m of all non-permafrost soils would be similar in magnitude to the annual global net atmospheric CO_2_ growth^5^. The second initiatives were the Koronivia workshops on agriculture, which included soils and SOC for climate-change mitigation and were initiated at COP23 in 2018. Finally last year, the FAO launched RECSOIL, a program for the recarbonization of soils^6^. The message of all three initiatives is complementary and simple: increasing SOC can partly mitigate carbon emissions and is, at the same time, indispensable for the adaptation of agricultural systems to climate change due to the numerous co-benefits it offers. SOC has positive effects on soil structure, water retention, and nutrient supply, and is crucial to sustain ecosystem services and agricultural productivity.

Political and market support are needed to motivate farmers to adopt sustainable agricultural practices on a scale large enough to result in the transformation of agricultural production systems. Messages that are easy to convey are needed to engage with policymakers and practitioners. Thus, the intent of the 4p1000 initiative was to be simple and easy to communicate. The goal is aspirational in that it is not viable for all land uses, soils, and all regions^7,8^. It is also an inspirational target, designed to raise awareness of the need to improve soil health and food security with opportunities for climate-change mitigation^9^.

The implementation of SOC sequestration on a large-scale is complex, as it involves different soil groups (defined by an IUSS Working Group^10^) and their specific management in different climate regions of the world. It will therefore need diverse tailored approaches. To achieve the required major changes in land-use practices, actions have to be supported by strong scientific, educational, political, and social programs that rely on multistakeholder interactions and transdisciplinary collaboration^9^. What those are, and how they would be instituted, remains the critical issue moving forward.

We identify region-specific opportunities for C sequestration as linked to both restoration of degraded soils and related improvement of crop yields. To gain and maintain SOC under climate change, we have to increase C inputs. We highlight that this is most easily communicated at sites where soils have both the largest C debt and where yield gap is high. At sites with low C debt, organic matter probably plays only a minor role in closing yield gaps. The identification of priority regions is supported by soil group as a basic mapping unit, which integrates relevant properties controlling yield and C storage, whereas the C-rich organic soils must remain protected. Hence, this paper offers a soil-specific perspective on feasible C sequestration and some of its trade-offs, which both depend on regional soil conditions, regional biomass availability, and, importantly, on regional social, economic, and political constraints.

## Linking soil C sequestration to food security as the way ahead

**Fig. 1:**
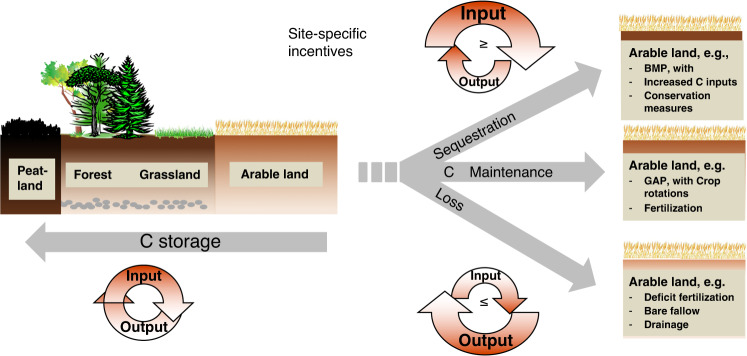
Conceptualization of C sequestration potentials in arable land. Usually C is lost after land-use conversion from native ecosystems (e.g., peatlands, forests, grasslands) to arable land. Future C storage in agricultural fields then depends on agricultural management practices, with options to regain C by increasing the organic matter input relative to ongoing CO_2_ release at best management practice options (BMP), to maintain C stocks by continued good agricultural practice (GAP), or to lose additional C by intensifying agriculture without additional C input, usually followed by soil degradation.

Any ton of CO_2_ that plants assimilate and that is subsequently sequestered in soil has been removed directly from the atmosphere and will thus help to mitigate climate change (Fig. 1). The science of CO_2_ sequestration in soils is currently advanced enough to inform the creation of policy and incentive programs despite some uncertainty in the absolute sequestration rates of particular practices in specific places^11–13^. To be successfully implemented at a global scale, appropriate SOC sequestration management strategies are likely to be adopted faster if SOC  is considered not only as a means for mitigating climate change but also as a contributor to soil health, increased food security, and other sustainable development goals^14–16^.

Currently, 33% of the global soils have been degraded^6^ and have lost much of their SOC through the historical expansion of agriculture and pastoralism^17^ and subsequent land-use conversion from native ecosystems (e.g., peatlands, forests, grasslands) to arable land (Fig. 1). This has resulted in a decline in soil structural stability, increased erosion risks, and reduced water storage and nutrient supplies. Soil degradation has thus become a major threat to food security, especially in developing countries^18^. Soil degradation can proceed when intensifying agriculture without additional C input. Soil degradation can be stopped with the maintenance of SOC stocks at good agricultural practice (GAP). However, by increasing the organic matter input relative to ongoing CO_2_ release at best-management practice (BMP) options as, e.g., outlined in the 4p1000 flyer, soil degradation can be reversed by increasing SOC stocks^12^ (Fig. 1). The related soil-health benefits from sequestering carbon may then help to close yield gaps in arable soils due to associated improvements in nutrient supplies, water-holding capacity, and soil structural stability^19,20^. Oldfield et al.^21^ reported that building SOC has the potential to close 32% of the global yield gap for maize and 66% of that for wheat, while also reducing fertilizer needs by 5–7%, respectively. Closing the yield gap would also reduce the need for further agricultural expansion and associated potential SOC loss^12^. To achieve these benefits, priority for the transformation of agricultural systems to increase SOC sequestration should be given to regions with large yield gaps, e.g., up to 90%, sub-Saharan Africa, and South and West Asia [www.yieldgap.org]^22^.

Priority for the transformation of agricultural systems to increase SOC sequestration should also be given to regions with low SOC contents caused by large historic SOC losses^17^. Unfortunately, the total area of degraded soil, ranging from 1000 to 6000 M ha^−1^, is not well-defined globally^23^, thus impairing a global agenda that can target land restoration and thereby support climate mitigation.

Yield gaps and historical SOC losses vary across regions, therefore biophysical sequestration potentials cannot be achieved to the same degree on all soils in all ecoregions. This is also due to site-specific nutrient requirements, which limit C sequestration in non-agricultural systems^7,8^, and which may cause trade-offs with nitrate release and particularly nitrous oxide emissions^24–26^. The analysis of yield gaps and soil C  debt in tropical and temperate soils (424 sites from 38 countries) shows that there is no direct relation between both parameters (Supplementary Fig. 1 and Supplementary Data 1), reflecting that the limitations of water and nutrients, in addition to SOC loss, are the major yield-limiting factors.

## Options for measurement and verification

Soil management is highly decentralized, and to a large extent, under the control of individual landowners. Additional research is needed to accurately predict C sequestration potentials at farm-scale resolution and for different soil groups, while also accounting for historical land management and past sequestration success^27^. In particular, it is not currently feasible to verify sequestration rates that increase total SOC stocks by <1% on an annual basis using direct soil measurements^15,28^. Considering the general agreement that the most effective way to accumulate SOC is to increase C inputs^29,30^ (Fig. 1), we may possibly overcome the desire of continuous assessment of changes in labile or stable SOC pools^31^. In other words, soils can remain at high organic carbon levels as long as improved management and C input are retained, irrespective of which form this carbon exists and how it is stabilized. We can thus promote shifts in management toward higher C inputs because the additional net amount of SOC sequestered will reside in soil until a new equilibrium is reached, which can take a few decades^32^.

It remains difficult to evaluate what quantitative contribution sequestered C finally makes to improving crop yields, and therefore to persuade regional policymakers to implement incentives if they cannot control the success of these measures^28^. In the search for a solution, some considerations exist to couple policy making with measurement and monitoring technologies on broader aggregate regional or global scales^33–35^, and to reward the C storage achieved to date, through so-called moving baselines^36^. Analytical tools to assess C storage exist and have been extensively discussed in the literature^17,28,36–40^. In particular when evaluated regionally against benchmark sites, such analytical tools provide clues to the potential long-term success of C- sequestration measures in the different soils of the world.

## Region-specific potentials and opportunities for soil carbon sequestration

Potentials to sequester C in soil show substantial variation from one region to another, even under the same type of management, due to variations and gaps in current and potential SOC levels^41,42^. Variations in C sequestration potentials increase with differences in climate, soil groups, cropping systems, and available technologies as well as with different yield gaps (www.yieldgap.org) and soil-specific, historical C losses^43^. This unfortunate reality can be a barrier to the global implementation of a soil carbon climate-mitigation initiative, which will thus need a coordinated effort at regional scales adapted to these variations. Putting such region-specific potentials for C sequestration and/or loss into action requires actions with regard to finalizing yield gap maps and land-degradation maps and providing them at high resolution, obtaining relevant soil information for many regions of the world, and validating methodologies for measuring SOC and its effect on soil functions across farming systems, ecoregions, and country borders.

The most pressing need is the development of an agenda that includes (i) information on region-specific soil distribution and degradation status, (ii) matching of sustainable management practices to soil group and its degradation status, and (iii) stopping the C loss from specific soils that have the potential to significantly affect the global C balance, e.g., peatlands under drainage. Currently, only a few countries have robust monitoring, reporting, and verification systems, but there are ongoing research efforts to expand these capabilities^28,36^. Global harmonization of data acquisition as already initiated by FAO’s Global Soil Partnership (http://www.fao.org/global-soil-partnership/en/, e.g., Pillar four) and data sharing needs to be urgently intensified to provide science-based information on soil status and its response to climate change.

For (i): A soil group as defined in the World Reference base^10^ already integrates information on basic properties relevant for soil fertility, such as pH, texture, cation exchange capacity, or hardpans. Different Reference Soil Groups also have different C storage potentials^44^, due to soil group-specific mechanisms of SOC stabilization^45^. This information should be utilized to guide the selection of priority areas for C sequestration and respective management.

In some west European countries, soils are already mapped at high resolution (e.g., 1:5000 for many areas of Germany, 1:20.000 in Belgium, 1:50.000 in the Netherlands and in France), and digital soil mapping should improve coverage. In these areas, SOC levels and soil degradation status are usually known; yet, the challenge is that this information cannot be utilized for a centralized climate-mitigation strategy due to restricted data access. Political will is needed to overcome such data protection issues when implementing large-scale SOC sequestration programs.

In developing countries, in contrast, such maps are frequently lacking or at a scale too coarse (e.g., 1:1,000,000 for Zambia) to infer site-specific management options from regional-scale map grids. The frequent lack of reliable, detailed maps of the state of soil degradation in these areas^23^ makes it difficult to manage the link between food security and soil restoration through C sequestration. However, this link can be established if it is supported by local incentives for farmers and stakeholders, who are usually well informed about the status of their soils.

For (ii): matching sustainable practices to soil group and degradation status, two general soil categories have to be distinguished: mineral soils containing only a few percent of organic C, but which cover more than 90% of the landsurface, and organic soils that are rich in organic C, such as peatlands and wetlands, but which cover only 3% of the landsurface^46^ but store more than 20% of all soil organic C^47^. The management practices to be applied to these two categories are expressly different^9^. In general, actions to increase SOC sequestration are focusing mainly on mineral soils, while the objective for organic soils is to reduce SOC loss.

Practices that retain and increase SOC stocks in agricultural soils are well established^13^, but may require more action to implement them. Many of these practices relate to known best-management practices to improve food security^48,49^. In China, for example, available best-management practices could attain net SOC sequestration equivalent to one-third of the potential for agricultural soils^50,51^. The proposed measures will, in particular, sequester C where the soils have lost SOC in the past. To gain and maintain SOC over time, we thus have to increase and maintain C input (Fig. 1), at best via increased crop-residue return and maintenance of increased yield.

The practices to be employed should be soil group-specific, accounting for their actual degradation status, as both aspects affect C storage potentials^44,45^. The contrasting physicochemical properties of these soils can be utilized to define management needs for SOC sequestration in the respective regions where these soil groups dominate. Examples of adequate practices for target major reference soil groups are given by Driessen et al.^52^ and outlined in Box 1, thus also highlighting target regions where these soils are most abundant (see Box 1 and Supplementary Information for more details). To restore degraded soils, specific management options usually have to be combined. When soils are little if at all degraded and yield is already at optimum, the potential to sequester additional SOC sustainably is limited. It should be kept in mind that higher inputs might be needed in these soils under climate change to maintain actual C contents.

Even with soil-specific management, global yields and thus the closure of yield gaps for sequestering C in soil rely on the addition of fertilizers, particularly of N. Linking C sequestration to food security thus requires sustainable management of N by relying, e.g., on site-specific fertilizer recommendations, as well as on other means such as organic residue return and the use of legume plants to fix atmospheric N wherever possible. A soil group-specific evaluation of these chances and risks is still lacking.

Measures that are less dependent on additional N storage are options of using biochar within the value chain for sequestering C and improving yields (e.g., refs. ^53,54^, with potential co-benefits for, e.g., N efficiency (see Supplementary Information). Yet, also these options are likely soil and crop and possibly even management specific^54^. Hence, for biochar application as well as for other options indicated above (Box 1), soil-specific differences require local implementation advice.

Due to the size and fragility of the carbon stock in wetlands and peatlands, (iii) SOC losses must be stopped from these soils, i.e., their management merits separate attention in carbon-sequestration-focused efforts^55–57^. Between 1850 and 2015, c. 50 Mha of peatlands have been drained, half of it for agriculture, with the released 80 Gt CO_2_-eq. Towards the end of the century, cumulative emissions from drained peatlands without management change may reach 250 Gt CO_2_-eq^58^. Whereas historically, most emissions were derived from the temperate zone, drained tropical peatlands, particularly in SE Asia, contribute the major part of current emissions^59^.

Upon rewetting, however, these emissions can be substantially lowered^60^ and eventually even reversed^61^. Rewetting might occur at the expense of producing food, fiber, and bioenergy crops and may require enhanced food production in other areas. Yet, the area of organic soils currently used for agriculture of c. 25 Mha is <1.5% of the global cropland and managed grassland area. Taking managed peatlands out of production might be a viable approach to meet multiple targets of climate mitigation, combating biodiversity loss, and restoring regional hydrology. Sparing peatlands for natural regeneration could be compensated by closing yield gaps^62^.

Box 1: Soil-specific options for carbon sequestration (exemplarily)

**Table:** 

Measure	Potential effect	Major target soil group for C sequestration	Regions with a high abundance of target soils (examples)
Fertilizer and organic residue management	Improved fertility and related crop yield and crop-residue inputs of soils that are: - Poor in nutrients, - Poor in SOC, or - Saline	Soils at subsistence farms, especially in low crop yield regions, e.g., - Highly weathered soils (Acrisols, Ferralsols, Lixisols) - Sandy soils (Arenosols) - Semiarid soils (Calcisols, Gypsisols, Solonetz, Solonchak)	- Subsaharan Africa - Brazil - Australia - NW-China
Liming	Improved fertility and crop-residue return in acidified soil; reduction of N_2_O release	Common soils under the agricultural practice that are acidic in the topsoil, e.g., Cambisol, Luvisol, Phaeozem, Acrisol, Ferralsol, Andosol	- China: acidified areas under intensive N fertilization - Subsaharan Africa, Brazil
Biochar application	Improved soil physicochemical conditions Reduced N_2_O and NH_4_ emissions	Highly weathered soils (Acrisols, Ferralsols, Lixisols, Podzols), and tropical Arenosols Waterlogged soils under rice cultivation	- Brazil, Columbia, - S- and E-Asia
Mulching (e.g., with no-tillage practice) and cover cropping	Enhanced SOC input Cooling, moisture preservation, erosion protection, prolongation of cropping period in the tropics and subtropics	Soils with large C debt Soils affected by heat, e.g., Acrisols, Ferralsols, Lixisols, Nitisols of the tropics and subtropics	- Ethiopia, N- and NW-China
Soil management, e.g, bed and furrow management, and reduced or no-tillage	Less SOC decomposition by reducing aeration and protecting the soil structure	Soils affected by waterlogging (Vertisols), and erosion (e.g., Phaozems, Chernozems, Ferralsols, Acrisols; sloping soils)	- India, Ethiopia, USA, Russia, Brazil, Zaire, China
Deep soil loosening, deep soil inversion, clay devolving	Subsoil C incorporation with enhanced yield	hardpans (by management: Anthrosols, Luvisols, Durisols)	- E-Europe - New Zealand, Australia
Crop systems management, e.g., precision farming, use of crop mixtures, cover crops, combined farming like rice-shrimp	Optimization of resource use and SOC sequestration in spatially heterogeneous landscapes, or at temporally varying water supply	Abundant arable soil groups - (e.g., Luvisols, Cambisols, Acrisols) - Fluvisols	- W-Europe, Australia - Vietnam, S-China
Water management, e.g., - Flooding	Reduced SOC decomposition by rewetting and avoiding drainage	Temporarily flooded soils, such as paddy rice systems (Anthrosols) or near rivers (Fluvisols); peatlands and organic soils (Histosols), soils with groundwater (Gleysols) or stagnant water (Stagnosols, Planosols)	SE Asia, central Africa
- Irrigation	Increase yields in dry areas, manage salinity when drained	Fertile semiarid soils (e.g., Kastanozems, Lixisols), salt-affected soils (Calcisols, Solenetz)	Southern USA, South Africa Australia

Reasoning given in Supplementary Information soils classified according to Word Reference Base^10^.

## Economic and political opportunities to achieve widespread adoption of soil carbon-promoting management measures

Financing gaps exist to sequester climatically significant amounts of C in soils, especially in developing countries. Even if funds can be provided, they must be accompanied by the development of institutions and processes that can support such investments. This is especially relevant in countries that are politically unstable and lack robust financial and regulatory institutions, such as some regions of the tropic and subtropics, where the need for yield increase with related soil C sequestration is greatest. In North America, Australia, and Europe, such institutions exist, but despite this, these regions have not sequestered climatically significant quantities of C to date.

An additional issue is estimating the change in the value of co-benefits, such as the promotion of biodiversity or regulation of the water cycle, erosion reduction, or other societal benefits as a result of soil management changes^63–65^. These co-benefits also vary spatially and temporally and generate a range of private values for individual farmers who create them and for society as a whole that also obtains public value from these benefits.

At present, SOC has not been successfully featured into market-based policies, for two overarching reasons: (1) payments for ecosystem services (PES), including C sequestration in soil, are rarely concrete as the benefits are difficult to measure and not standardized, thus requiring mediation between global beneficiaries and local and regional service providers. (2) Individual land managers do not focus on sequestering C but on agricultural production. Therefore, it is necessary to create additional incentives for farmers to sequester additional SOC, such as identifying enhancements in productivity, superior market access, or financial returns to carbon assets^66^.

Net cost estimates for changing management practices to increase SOC range from $3/ton CO_2_ to $130/ton CO_2_^67^. They are influenced by soil-specific management change and related ability to increase SOC at a given site (Box 1), i.e., these costs vary considerably across regional scales. Nevertheless, incentives to adopt management changes that sequester additional C have a history of some success, either created by the public or private sectors (or both), for example, in Australia^66^. Potential incentives include subsidies, taxes, and market-based payments for carbon or cap and trade systems, the right choice depending on regional or national politics, societal preferences, and implementation costs^13,35^. Each of these options deserves further scrutiny for their suitability to lead to large-scale SOC sequestration.

Social norms as well as psychological and behavioral factors need to be considered for widespread adoption of soil carbon-promoting management measures^13,68^. These uncertainties and complexities make a regional and particularly a national soil management strategy for carbon sequestration a so-called wicked policy issue, with multiple potential avenues^69^. As a wicked problem, it cannot be solved with single policy action. In this context, it is crucial to identify strategies that meet multiple goals by linking soil C sequestration and greenhouse gas-emission reductions to food security, biodiversity, and environmental quality. The solutions that are achievable are likely to be diverse and incremental. There will be no single global “silver bullet”, but rather a vast array of small, diverse, and hopefully interconnecting “silver buckshot” policies^70^.

## Tasks and agenda for implementation

There is a general agreement that the most effective way to accumulate SOC is to increase C inputs (Fig. 1 (eg., refs. ^29,30^); organic substrates fulfill this purpose only partly if at all, because meaningful increases of carbon sequestration at one farm or region must occur without simultaneous reductions in SOC at another location from where this material is transported from. Hence, organic C inputs into soil must be produced on-site, i.e., by enhancing crop production and green manure^71^.

Based on the challenges to develop a global agenda, we suggest to focus on seven main points of research and development (R&D) that can support local or region-specific SOC sequestration schemes (Figure for Box 2; see “need to know” criteria), with six additional research foci that would help to further advance the agenda of a global soil–climate-mitigation strategy (figure for Box 2; “nice to have” criteria, see Supplementary Information for more detailed reasoning). Focusing on yield gaps is one option to make implementation feasible because it is transparent and accepted by farmers. However, it may not be the tool to maximize overall C sequestration, due to weak direct global correlations with the yield gap (Supplementary Fig. 1 and Supplementary Information, see also^21^. For the latter, we have to consider overall and soil-group-specific C sequestration potentials (figure for Box 2, right), as well as alignments to other co-benefits, such as biodiversity, regulation of the water cycle, and specific country needs, e.g., biowaste recycling in China.

In as much as yield gaps relate to soil degradation, any means to restore soils will increase yields, crop-residue return, and therewith contribute to sequestering CO_2_ from the atmosphere. These links, though logically well understood, are difficult to quantify at high spatial resolution. We identified the following seven urgent needs to make a global soil mitigation strategy successful (figure for Box 2):

i. *Improve soil and yield gap information systems for different regions of the world:* Currently, soil degradation maps are not reliable^23^ and frequently not available at a regional scale. The yield gap atlas (www.yieldgap.org) does not yet include all countries. Soil maps, when existing, are frequently provided at a resolution too coarse and thus not good enough for regional soil management and for informing farmers about how to optimize SOC management at the local level, especially in many tropical and subtropical countries such as sub-Saharan Africa.
ii. *Reliable predictions of local and regional yield development per tons sequestered C*: The main potential for significant carbon sequestration lies in the world’s arable soils with large yield gaps due to low availability of nutrients and/or organic matter. To help farmers adopt SOC sequestration practices, information on the response to yield enhancement through increased SOC in different regions of the world is crucial. Improved agricultural yields due to increased SOC were first reported by Lal^72^ and Pan et al.^20^. Recent meta-analyses suggest that yield increases flatten out at 2% SOC^21^. Global maps with region-specific yield responses related to SOC increases therefore will greatly support the efficient implementation of sustainable SOC sequestration practices.
iii. *Additional fertilizer requirements for sustainable C sequestration*: Soil organic carbon sequestration frequently requires large amounts of mineral fertilizers^7^, in particular to replace nutrient removal with harvest and/or to increase the fertility of degraded land^73^. As fertilizer production is energy consuming, however, we should never aim at maximizing C sequestration by maximizing N or other nutrient inputs. Instead, fertilizer input should be synchronized to plant uptake and site-specific, pedoclimatic conditions to prevent losses into the gas phase or contamination of water bodies^74,75^, as well as to reduce costs.
iv. *Full life-cycle greenhouse gas accounting within C sequestering farming systems:* To be able to evaluate the efficiency of soil C sequestration measures, their impact on other greenhouse gas emissions has to be taken into account. This may concern machinery use and transport as well as other greenhouse gas emissions during, e.g., fertilizer production. Life-cycle analyses have been applied for biochar use as a negative emission strategy^53^. They are particularly necessary with regards to N fertilizer use, which inevitably generates N_2_O emissions with a global warming potential exceeding that of CO_2_ by a factor of almost 300, depending on the time horizon under consideration^26,76^. Relatively modest increases in N_2_O (or CH_4_) emissions could partly, or even completely, offset any reductions in atmospheric CO_2_ resulting from increased soil C storage^24^.
v. *Assessment and regional mapping of soil C sequestration:* While incentives for farmers may promote C sequestration at farm scale, policy advice at regional or national scale requires larger scale predictions of possible SOC-sequestration potentials by simple analytical tools or modeling approaches^28,36,77^.
vi. *Accounting* “*off-site*” *transfers of organic amendments, e.g., manure, compost, biochar, to the soil at a state or country level:* A meaningful increase of C  sequestration at one farm or region must occur without simultaneous reductions in SOC at another location. In the carbon market, this option is known as “carbon leakage”, and its consideration is an important pillar of mitigation policy. This could be the case, e.g., by the use of manure or compost that is bought and transported from another region where it is not in excess and also needed for maintaining or improving C storage and soil fertility. Data collection could be done via declaration forms; for instance, German farmers complete such a form when they apply farmyard manure, including the origin of the manure used.
vii. *The Broad ensemble of policies and bottom-up approaches including farmers’ incentives, societal standards, and actions to scale up adoption of C sequestering practices*: The overall social, economic, and cultural challenges of changing management toward soil C sequestration should be addressed through a diverse set of incentives and measures. They must take into account region-specific barriers that may hinder the implementation of C-sequestration practices in soil, such as security of tenure, lack of financial resources, or gender equality^9,78^.

Government farm subsidies represent a significant source of support for global farming, conservation, and other related activities, currently totaling an estimated US$445 billion per year^79^. Some of these expenditures could be focused on sites and activities most beneficial for climate-change mitigation. We suggest to start identifying priority sites based on indicators for C sequestration potentials and then comparing these with yield gap analyses or vice versa (figure for Box 2, right scheme), followed by soil-specific amelioration measures. Yet, low yield gaps should not necessarily prevent action, because there may also be high C sequestration potentials on fertile lands if the actual yield gap is low, and because the achievements of co-benefits like biodiversity, water storage and resilience may change priority setting (see also Supplementary Table 1, Supplementary Information, for an example of a more detailed site prioritization).

A related scheme for site prioritization could potentially be applied to any country, focusing on the specific soils and yield gaps of region-specific crops of the region, with paddy rice, for instance, in SE Asia, wheat in Austalia and Europe, or maize in North America and Africa. As an example, Table 1 illustrates such a case scenario for water-limited yield gaps for maize at three sites in Zambia (see Supplementary Data 1 for individual data collection). Consultation from local soil scientists and agricultural officers or even targeted field surveys may be needed to derive soil group and specific degradation status. A related success story is the Farmer Input Support Program of Zambia, where the government supports efficient soil fertilization by farmers via an E-voucher system, which increases crop-residue return and may thereby increase SOC, even at small-holder farm level (http://www.pmrczambia.com/wp-content/uploads/2015/09/Farmer-Input-Support-Programme-Infographic.pdf). Soil scientists should document this success and coordinate long-term field experiments across the globe^80,81^. Moreover, soil scientists should also join forces to harmonize the different initiatives to map yield gaps, soils, and soil degradation status around the world. Any success stories, even small and local ones, can serve as templates for implementation.

**Table 1:** Breaking down a global soil–climate-mitigation strategy to regions.

Site	Yield gap (%)	C debt (t ha^−1^)	Soil group	Soil degradation	Recommended climate mitigation action	Expected change in economic revenue	Expected carbon sequestration
Living stone	84	3	Leptic Acrisol	Nutrient loss	None	++	−
Kasame	84	16	HaplicAcrisol	Nutrient loss	Liming + fertilization; conservation agriculture	++	++
Choma	75	22	Leptic Acrisol	Nutrient loss	Conservation agriculture and agroforestry	+	+++

Examples from three sites in Zambia (data sources: www.yieldgap.org, and ref. ^17^ showing water-limited yield gap for maize and C-debt analyses.

The number of plus/minus symbols illustrates possible success/failure for yield improvement and C sequestration when investing into soils, respectively.

It is critical to be aware that soil stewardship cannot be expected to alleviate all socioeconomic factors that impose a risk for investment and low yields. However, linkage to yield gaps can be easily communicated and thus facilitate acceptance by policymakers and farmers. It may circumvent the problem of persuading policymakers to implement incentives for soil C sequestration if they cannot measure whether it has happened after a reasonable timescale^28^, e.g., after 1 or 2 years. If not solely focussing on the amount of SOC sequestered itself but on closing yield gaps to restore formerly degraded soil, such focus can be more easily communicated, measured, and even re-finance investment schemes. This will lead to more organic C being introduced into the soil through best-management practices that are adjusted to the specific soil condition. The site-specificity of best-management practice justifies an agenda of diversification. To achieve this, we need to consider data protection issues in using localized soil information for climate-mitigation measures and regulating excessive N inputs in order to reduce trade-offs from N_2_O release and nitrate pollution.

The greatest current impediments to introducing sustainable soil management practices are the absence of adoption incentives. However, it is already relatively common, in some nations, to tie compliance with conservation goals to price support or to crop insurance payments^82^. This can be (and has been) a successful method for improving soil management. Orientating such policies on a combination of increasing crop yields where needed and soil-specific C sequestration potentials and amelioration measures can break down a global agenda for collaborative and successful action to the regional scale. Linking C sequestration in the soil to programs on food security and poverty alleviation in rural areas, soil health, and REDD + (reducing emissions from deforestation and forest degradation) and biodiversity might facilitate further policy development and accelerate implementation. Aligning UN conventions for climate change, biodiversity, and land-degradation neutrality would further reduce overlapping organizational efforts and accelerate the identification of regional priority areas. Moreover, it could help to bring SOC management closer to the heart of many important societal issues.

An example demonstrating that joint coordination can facilitate success is the organic standard, which refers to worldwide standards and certification issues in the organic food sector (https://organicstandard.com/). This standard helped formalize the science–policy dialog, and engaged civil society into a discourse on sustainability and well-being^83^. This approach might also perform well in terms of C sequestration and associated conservation measures^84^, thus also supporting the achievement of other sustainable development goals.

Social and economic measures to implement C sequestration programs quickly and effectively include partnerships between business and the NGO sector. Particularly noteworthy are the activities of several large companies, such as Coca-Cola, Mars, Repsol, Fronterra, Walmart, or DANONE Inc, who have in response to consumer demand committed to significant emission reductions and actions which will lead to increased sustainability in agricultural systems. DANONE Inc, for example, is embarking on a program to become C neutral by 2050 (https://www.danone.com/impact/planet/towards-carbon-neutrality.html). Due to the scope and size of the corporation, the company may be able to influence farm management programs for an area about half the size of Belgium. Even though the programs may not sequester C at the desired quantity to offset global emissions, they may inspire further innovations once investments occur and new corporate policies are implemented by producers. Future policy action could include innovative structures that recognize the benefit from engaging with agribusinesses and related industries with supply-chains that have a land management component. An additional advantage of such a structure is the potential for more effective integration across and beyond geopolitical boundaries.

All the approaches we have suggested rely on multistakeholder collaboration. Even if orienting on soil-group-specific measures, the steps towards the global-scale soil–climate-mitigation strategy are therefore diverse, imperfect, incremental, and take time. However, they provide an opportunity because they are based on the real mechanisms to convert science into practice.

The future gives hope: through the 4p1000 initiative, FAO RECSOIL, and the Koronivia workshops on agriculture, we have laid the foundations for moving from discussion to opportunities to create sustainable solutions. A soil–climate-mitigation strategy will be globally successful if it takes soil-specific aspects into account. We can identify priority areas where soil organic C storage can improve soil fertility and crop yields to motivate farmers while excluding regions that can likely be disregarded because of negative trade-offs. However, future policy measures should (1) take into account the benefits of engagements with agribusinesses and related supply chain management industries that have a soil management component, (2) seek to encourage joint small-scale actions involving local actors from and across the border regions, and (3) improve local capabilities for sustainable site-specific soil management. These efforts are likely to be able to work more effectively across geopolitical boundaries, and to tackle the common task of soil–climate-change mitigation on a scale and at a level suited to the complex challenge of land management.

Box 2 Science R&D crucial to support the global implementation of C sequestration (need to know) and to further advance the agenda (nice to have)

**Table:** 

${\bf{{Need}}} \, {\bf{{to}}} \, {\bf{{know}}} \, {\bf{{(supportive}}} \, {\bf{{science)}}}$	${\bf{{Agenda}}} \, {\bf{{for}}} \, {\bf{{priority}}} \, {\bf{{site}}} \, {\bf{{selection}}}$
1. Detailed soil information systems for different regions of the world that include assessments of yield gaps and soil degradation status	
2. Reliable predictions of local and regional yield development per ton sequestered C	
3. Additional fertilizer requirements for sustainable C sequestration.	
4. Full life-cycle greenhouse gas accounting in C sequestering farming systems	
5. Detailed maps of regional and national soil C sequestration potential.	
6. Accounting for transfers of organic material that may reduce stored carbon elsewhere	
7. Broad ensemble of policies and bottom-up approaches including farmers’ incentives, societal standards and actions to scale up adoption of C sequestering practices	
**Nice to have**	
1. A priori assessment of regional C sequestration potentials	
2. Quantitative estimates on the persistence of sequestered soil C in different regions	
3. Evaluation of subsoil storage options for additional C	
4. Closing gaps in terrestrial soil C modeling, e.g., by including erosion or fate of inorganic C and dynamics of peatland carbon in natural and drained state	
5. Harmonized, simple analytical tools for a priori assessment of legacy-driven C losses and C sequestration potentials	
6. Documentation of C sequestration success stories for different soils and climatic regions, including monitoring of ecosystem services and societal benefits

## Supplementary information

Supplementary Information

Supplementary Data 1

Description of Additional Supplementary Files
