# Supplementary material for: Towards a global-scale soil climate mitigation strategy
Source: Nat Commun. 2020 Oct 27;11:5427. doi: 10.1038/s41467-020-18887-7 (PMC7591914; doi:10.1038/s41467-020-18887-7)
Supplement: Supplementary file 1 — Supplementary Information [file 41467_2020_18887_MOESM1_ESM.pdf]

Supplementary Information to:

Towards a global scale soil climate mitigation strategy

W. Amelung[1,7*], D. Bossio[2], W. de Vries[3], I. Kögel-Knabner[4], J. Lehmann[5], R. Amundson[6], R. Bol[7], C. Collins[8], R. Lal[9], J. Leifeld[10], B. Minasny[11], G. Pan[12], K. Paustian[13], C. Rumpel[14], J. Sanderman[15], J.W. van Groenigen[16], S. Mooney[17], B. van Wesemael[18], M. Wander[19], and A. Chabbi[20*]

[1] Institute of Crop Science and Resource Conservation – Soil Science and Soil Ecology, University of Bonn, Germany.

[2] The Nature Conservancy, Arlington, VA, USA.

[3] Wageningen, University and Research, Environmental Research, 6700 AA Wageningen, The Netherlands.

[4] Chair of Soil Science, Department of Ecology and Ecosystem Management and Institute of Advanced Study (TUM-IAS), Technische Universität München, München, Germany.

[5] Soil and Crop Science, School of Integrative Plant Science Cornell University, Ithaca, NY, USA. And Institute of Advanced Studies, Technical University Munich, Garching, Germany.

[6] Department of Environmental Science, Policy, and Management, University of California, Berkeley, CA 94720, USA.

[7] Institute of Bio-and Geosciences, Agrosphere (IBG3), Forschungszentrum Jülich GmbH, Jülich, Germany.

[8] Department of Geography and Environmental Science, University of Reading, UK.

[9] Carbon Management and Sequestration Center, FAES/SENR, The Ohio State University, Columbus, OH 43210, USA.

[10] Agroscope, Climate and Agriculture Group, 8046 Zurich, Switzerland

[11] School of Life and Environmental Sciences, Sydney Institute of Agriculture, The University of Sydney, NSW 2006, Australia.

[12] Institute of Resources, Ecosystem and Environment of Agriculture, Nanjing Agricultural University, Nanjing 210095 China.

[13] Department of Soil and Crop Sciences and Natural Resource Ecology Lab, Colorado State University USA.

[14] CNRS, Institute for Ecology and Environmental Sciences (IEES) Paris, France.

[15] Woods Hole Research Center, Falmouth MA 02540, USA.

[16] Soil Biology Group, Wageningen University, 6700 AA Wageningen, The Netherlands.

[17] O'Neill School of Public and Environmental Affairs, Indiana University, USA.

[18] Earth and Life Institute, UC Louvain, Louvain La Neuve, Belgium.

[19] Natural Resources and Environmental Sciences, University of Illinois at Urbana-Champaign College of Agriculture, Consumer and Environmental Sciences, USA.

[20] Institut National de Recherche pour l'Agriculture, l'Alimentation et l'Environnement (INRAE) Centre de Recherche Nouvelle-Aquitaine-Poitiers, (URP3F) Lusignan, France and UMR ECOSYS, Centre INRAE, Versailles-Grignon, Bâtiment EGER, Thiverval-Grignon, France.

Supplementary Figure 1

The relationship between yield gap (www.yieldgap.org) and soil C debt (data extracted from [1]) show significant variations for given C debts but no overall straight correlation, because other biogeophysical constraints rather than only SOC are limiting yields.

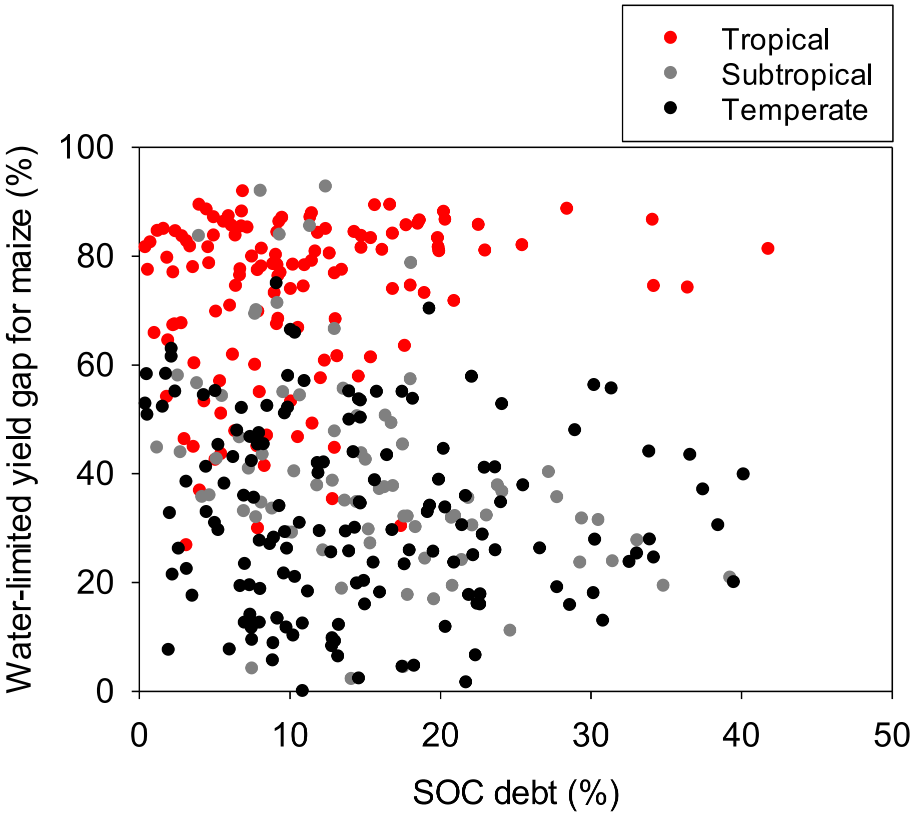

For each station where yieldgap.org [2; 3] has estimated potential yields and extracted actual regional yields for rainfed maize (n = 424), we extracted the soil organic carbon debt for those locations [4]. For illustration, sites were assigned to tropical climates at latitudes reaching 23.5°N or S; "subtropical" indicates sites with latitudes between 23.6 and 40°C at the Northern and Southern hemisphere, respectively.

Each station location is representative of an agronomical important unique climate x soil zone within a country. The mean size of this climate x soil zone is 93 km2 (25th - 75th quartiles = 12 - 103 km^2). The SOC debt was calculated at a roughly comparable resolution of 10 x 10 km.

Yield gap was calculated as: YG (%) = 100 x (YP - YA) / YP,

where YP (yield potential) was calculated using an appropriate crop simulation model assuming unlimited water and nutrients. Actual yield (YA) was the 10-year average of reported yields in that region for farmers following typical district practice.

Water-limited yield gap: YWG (%) = 100 x (YW - YA) / YW,

where YW (water limited yield potential) was calculated using a crop simulation model using actual rainfall at each station.

Supplementary Table 1.

Examples for site prioritization considering SOC sequestration potential, yield gap and soil degradation status. Note that this Table is meant as an example for priority setting, which may change with quantification of co-benefits from, e.g., biodiversity, water storage and ecosystem resilience.

Options	SOC sequestration	Yield Gap	Soil degradation	Priority
1	+	+	+	Very high
2	+	+	-	High
3	+	-	+	High
4	-	+	+	Moderate
5	+	-	-	Moderate
6	-	+	-	Low
7	-	-	+	Low
8	-	-	-	Low/None

Different major Reference Soil Groups (RSG) provide an important though yet underused guide to hotspots where SOC sequestration will be the most efficient. A RSG as defined by World Reference Base for soil resources (WRB) is based on specific and unique combinations of soil characteristics and properties that are relevant for both yield and carbon storage, such as cation exchange capacity, pH, texture, water holding capacity, and hardpans. This is more than the assessment of individual soil parameters, which may not all be available in maps, particularly not for the subsoil, and which alone do not determine how a particular soil may degrade or should be managed. The soil units within the RSG, on the other hand, can help to derive important yield and C-sequestration limiting factors, i.e. improved soil maps could help to steer possible large-scale management support, which would then need to be refined locally. Examples for such soil-specific means refer to Box 1 (main paper):

i) *Fertilizer and organic residue management* with high chance of improving soil fertility, e.g., by adding biomass C to SOC-poor soils (e.g. Arenosols), nutrients to nutrient-poor soils (e.g., Acrisols, Ferralsols, Lixisols), organic residues and gypsum to sodic soils (e.g., [5; 6]) and simply by adding nutrients in "unfertilized" soils [7; 8].
ii) *Liming* is commonly needed to adjust soil pH and thus to maintain high yields and crop residue return, in addition to potential positive effects on faunal activity and soil structure [9] and reducing N_2O emissions [10]. As reviewed by [9], positive effects of liming on SOC sequestration are not yet observed globally but reported for certain soil orders, like the acidic Ferralsols and Andosols with potential Al toxicity and some acidified sites in central Europe. Increasing soil pH may particularly enhance crop yields in regions where low pH constrains crop production, which is estimated to occur at 179 million hectares of arable land [11], being about 12% of the total crop land area. It is specifically relevant in non-calcareous soils with a low buffer capacity (CEC) that are receiving high N fertilizer rates, being the case in large parts of China [12; 13].
iii) *Biochar amendments* add persistent C to soil, aiming at both sequestering C and improving yields (e.g., [14]). Intriguingly, mere biochar additions showed variable effects on yields of some productive soils in humid climates [15; 16]. Biochar amendments resulted in positive

effects on soils when they were combined with inorganic fertilizers and organic manure additions irrespective of climate [17], in sandy and clayey soils with needs to improve aggregate formation [18; 19], and in some impressive improvements of the fertility of soils with pronounced anion exchange capacity benefiting from additional cation exchange by oxidized biochar surfaces (e.g., Ferralsols, Acrisols; [20; 21]). Biochar also helped to mitigate other greenhouse gas emissions, such as methane [22] and nitrous oxide [23] as well as to reduce ammonia emissions [24], thus helping to improve N use efficiency [25] at higher overall nutrient demands needed for yield gap closures.

iv) *Soil tillage*, e.g., adoption of reduced tillage or no-till options combined with mulching, particularly on Vertisols with potentially large C sequestration potentials due to elevated clay contents [26], and high C soils prone to erosion and benefiting from increased water storage under intensive cropping, like Kastanozems, Chernozems, Phaeozems [27]. These practices are also promising many soils in the tropics and subtropics like Acrisols and Ferralsols since they generally benefit from organically stored nutrients and in case of surface mulch also from lower soil temperatures, eventually allowing double or triple cropping, for instance. Much more radical methods like soil inversion, flipping or clay devolving may be needed to increase soil productivity and SOC levels in Durisols and other hardpan sites, like in Australia and New Zealand [28; 29], whereas soft methods like traditional subsoil composting can be beneficial for vulnerable sandy soils in arid climates [30; 31].

v) *Altered land-use systems*, e.g., use of precision farming and new field arrangements (e.g., multiple cropping systems) on abundant soil orders like fertile Luvisols with Cambisol associations and thus heterogeneous properties in the landscape, or even the combination of different management systems, such as of paddy management with duck, fish and rice-shrimp farming, for instance, on Fluvisols [32; 33; 34].

vi) *Water management*, e.g., protecting hotspots like peatlands [35] or any other managed organic soils may already positively affect future projections of net C effluxes. The same applies to the protection of carbon stock in mineral soils with high water tables (Gleysols, Stagnosols, Planosols; [36; 37; 38]. Protecting these soils from C losses may be achieved by flooding, or, simply, by avoiding ploughing and keeping them under grass or forest. In soils where water is limited or where yields are impaired by salts, adapted *irrigation* measured may be required.

It should be noted that management options are usually combined to maintain or improve soil fertility. The above-mentioned list is thus meant to illustrate that due to specific properties of different soil groups, different options to manage the soil have different potentials to close yield gaps and therewith to sequester C in the different soil groups. An exemplary first comprehensive guide to soil group-specific management options can be found in the appendix / lecture notes on the World Soil Reference Base [6], which can help policy makers in defining regional management potentials, but certainly require refinement on the ground, then also considering other factors such as length of cultivation, existing SOM levels, production system, farmer income or availability of inputs. Acrisols and Ferralsols, frequently found in the tropics and subtropics, for instance, require liming, are usually limited in P (same as Andosols), basic cations, and also N, and benefit from water storage and low surface temperatures with, e.g., surface mulch. Also, biochar shows most promises for such soil groups, as well as agroforestry, depending on the prevailing climatic conditions. Carbon sequestration in Vertisols must guarantee rooting when the soils have excess water when wet or when they are hard when dry, sometimes requiring specific bed and furrow management. Temperate soils like Luvisols, Chernozems and Phaeozems instead rather require adapted fertilization, maintenance liming, and reduced tillage protecting them from erosion. Arenosols lack soil aggregates and can thus not benefit from measures to improve soil

structure, but may benefit from sprinkler irrigation as commonly prone to drought. Similarly, calcareous soils do not require liming but may need, e.g., furrow irrigation.

Any additional nutrient input has to be done with care in order to avoid additional nitrous oxide release as greenhouse gas, if not reduced by temporary microbial N immobilization or nitrification inhibitors, the evaluation of the latter being beyond the scope of this study. Also, the rewetting soil in intensively used agricultural areas must be done with care to prevent excess formation of N_2O and CH_4, but to improve the full greenhouse gas balance of the system.

New ways to increase soil carbon contents may evolve, e.g., by using new plant cultivars that store more carbon in soil [39], or by growing algae on waste water-streams for C sequestration and organic fertilizer production [40; 41]. There is also a need for larger-scaled and more open, holistic crop rotations that include ley farming to enhance soil C storage and prevent its losses [42]. However, the success of such measures depends on site conditions and is thus region-specific.

As outlined in the main manuscript, main scientific tasks are now to: (1) Foster the establishment of a regionally relevant soil information system that contains localized information on soil group, degradation status, and yield gap. (2) Establish finely resolved maps showing C sequestration potentials in these regions and as related to yields in order to identify priorities and to define support schemes. (3) Improve predictive models for soil C sequestration as a function of site-specific nutrient requirements and management options in order to allocate resources effectively and to reduce risks when investing in C storage. (4) Develop sophisticated predictive models that include complete full life-cycle assessments of all greenhouse gas emissions on farm. These models are at best extended by closing remaining gaps in terrestrial C modelling, such as erosion-induced transport of SOC, fate of inorganic C, or the global black carbon cycle (see nice-to-have criteria). (5) Create regional and national scale maps of soil carbon sequestration potential. (6) Account for possible 'leakage' in carbon market terms, i.e. the transfers of organic amendments to a region from another, where it would also help to sequester C. (7) Provide a broad set of incentives and policy options to address the diverse, region-specific social and economic challenges in implementing C sequestration measures.

Additionally, we identified six nice-to have options:

i) *A-priori assessment of C sequestration potentials*: Estimates of SOC storage and SOC stabilization potential in agricultural soils at the regional to global scale need to be improved [43; 44]. Recently, first global estimates for C sequestration potentials have been provided for the terrestrial biosphere [45], although this requires further validation on regional numbers for the included measures and systems. With respect to agricultural soils, the resolution of such estimates is not fine enough to inform farmers on the optimization of SOC management at the local scale. One of the main reasons is the complexity of historic land use and management [46; 47]. In addition, past land may have left legacy effects on future SOC stock development irrespective of current land-use decisions [1]. As a result, declining SOC stocks despite current SOC conservational management may be observed [48; 49; 50]. Therefore, the development of scenario maps is critical for future SOC stock changes, based on historical data, underpinned with uncertainty ranges, and at best linkable with other similar joint efforts like global yield gap assessment.

ii) *Quantitative estimates on the persistence of sequestered SOC*: How stable is newly sequestered SOC remains a key valid criticism of SOC sequestration efforts. Only parts of the added labile Carbon are stabilized, e.g., via sorptive interactions of formerly dissolved organic C with mineral phases [51; 52; 53]. Another significant portion of additional biomass C input are sequestered in labile pools, because the whole process is controlled by C saturation [54; 55].

Unless C is added in stable forms such as biochar [14], a significant and still unknown fraction may be readily lost upon disturbance, and it will respond differently to unhalted climate change. The response reactions are likely non-linearly related to OM input [4]. Meyer et al. [56] reported that the response of soil C to warming exhibits significant regional variation. To provide reasonable numbers of longer-term C sequestration potentials, it is important to be able to relate potential C storage to potential C response reactions. Thus, it is essential to couple projections on future C storage to the valuing of its vulnerability to climate and management change.

iii) *Evaluation of subsoil storage options for additional C*: Up to 75% of total soil nutrient and carbon stocks may be stored below the plough layer [57; 58; 59], this has not yet even been considered in the 4p1000 initiative. Subsoils usually comprise old radiocarbon ages [60; 61; 62]. Nevertheless, additions of labile C and nutrients promoted mineralization of subsoil C [63; 64], which is also vulnerable upon land-use change [48; 59; 65]. Efforts to sequester C also in the subsoil by converting arable soil into, e.g., grasslands have been successful [66], but not always [67]. A promising alternative might be the direct residue placement into deeper soil, which may even support yield increases [68].

iv) *Gaps in terrestrial C cycle*: Despite recent advances in soil C turnover modelling, the coupling of these models to reduce current uncertainties in global C modelling still warrants further attention. Examples for such gaps are erosion-induced transport of soil carbon: With 36 Gt of sediments transported annually by world rivers, the fate of erosion-induced transport of SOC (along with complete accounting of CO_2, CH_4 and N_2O) must be considered in the global carbon budget [69; 70; 71]. Other examples relate to the modelling of the global cycle of black carbon and the incomplete residues of burning [62; 72; 73; 74]. Additionally, the impact of management on soil inorganic C (SIC) is not yet part of 4p1000, but globally, about one-third of total C is SIC [75]. Routine liming is recommended to sustain agricultural productivity, a typical rate is 4t $CaCO_3$ ha^{-1} in 3 years. This means that up to 360 kg SIC may be released as CO_2 per year, which can be larger as the potential C sequestration by no-tillage agriculture, for instance (324 kg C ha^{-1} yr^{-1}; [27]). Under paddy management, losses of SIC from parent material even exceeded the amount of SOC sequestered [65]. Gaps do also exist in incorporating organic matter dynamics of peatlands, both natural and drained, into modeling the terrestrial C cycle. Although models for natural Northern Peatlands exist [76]; dynamic modeling of greenhouse gases for managed peatlands is in its infancy. This is a major barrier towards implementing mitigation strategies given the often-observed radiative trade-offs between avoided CO_2 and accelerated CH_4 [77]. Also, the fate of CO_2 in soil is not yet clear, since large parts of CO_2 produced in the soil may not be immediately released to atmosphere but leached and thus at least temporarily stored in the regolith, amounting to c.0.1 Gt C annually [78].

v) *Harmonized analytical tools:* There is no method available to directly quantify the SOC sequestration potential in the field. Physical soil fractionation carried out to identify pools of different SOC stability and turnover time must still be adapted to the range of stabilization mechanisms that control SOC cycling in different major reference soil groups, e.g., as reflected in the different role of Fe and Al (hydr)oxides and phyllosilicates in the sequestering SOC in the tropics and temperate climate, respectively. Yet, such analytical procedures may provide early indicators of management impacts on SOC and may help to initialize and parameterize the underlying SOC models [79; 80; 81; 82]. To use these models for putting climate mitigation activities on the ground, however, there is still a need to combine them with longitudinal, long-term experiments, which allow full joint socio-economical and biophysical assessment.

vi) *Monitoring*: to convey farmers of the 4p1000 initiative, it must be a success story that is communicable via regional case studies within a global network approach [83]. For this purpose, there exists a need for well-coordinated large-scale monitoring studies at farm scale and rural areas for filling many of the data gaps in all regions, particularly in those currently underrepresented in global assessments. The monitoring should be specific for the different major soil groups and climatic regions. This monitoring system should likely also involve satellite imaging and spectroscopic soil C assessments [84; 85; 86; 87; 88], however, it should also be accompanied by on the ground surveys to obtain more hard data on the potential role of C sequestration for a range of other ecosystem services, such as biodiversity, water retention, and societal benefits in terms of improved livelihoods. The design of this global monitoring system and registry must be rigorous, verifiably and transparent, and at best underpinned by long-term experiments that include evaluation of social-economical and anthropological information.

References

1. Sanderman, J., Heng, T., Fiske, G.J. (2017a): Soil carbon debt of 12,000 years of human land use. Proceedings of the National Academy of Sciences, 114(36), 9575-9580. https://doi.org/10.1073/pnas.1706103114;
2. van Ittersuma, M.K., Cassman, K. G., Grassini, P.G., Pablo, J.W., Tittonell, P., Hochmand, Z., (2013): Yield gap analysis with local to global relevance—A review. Field Crops Research, 143, 4-17.
3. van Wart, J., Kersebaum K.Ch., Pen, Sh., Milner, M., Cassman, K.G. (2013): Estimating crop yield potential at regional to national scales. Field Crops Research, 143, 34–4.
4. Sanderman, J., Creamer, C. Baisden, W.T., Farrell, M., Fallon, S., (2017b): Greater soil carbon stocks and faster turnover rates with increasing agricultural productivity. SOIL, 3, 1-16 https://doi.org/10.5194/soil-3-1-2017.
5. Feller, C. , Beare, M.H. (1997): Physical control of soil organic matter dynamics in the tropics. Geoderma, 79, 69-116.
6. Driessen, P., Deckers, J., Spaargaren, O., Nachtergaele, F. (eds.) (2001). Lecture notes of the major soils of the world. World Soil Resources Reports 94, Rome: FAO.
7. Han, P., Zhang, W., Wang, G., Sun, W., Huang, Y. (2016): Changes in soil organic carbon in croplands subjected to fertilizer management: a global meta-analysis. Scientific Reports, 6, 27199.
8. Ladha, J. K., Reddy, C. K., Padre, A. T., van Kessel, C. (2011): Role of Nitrogen Fertilization in Sustaining Organic Matter in Cultivated Soils. Journal of Environment Quality, 40(6), 1756.
9. Paradelo, R., Virto, I., Chenu, C. (2015): Net effect of liming on soil organic carbon stocks: A review. Agriculture Ecosystems & Environment, 202, 98–107.
10. Shaaban M, Hu R, Wu Y, Younas, A., Xu, X., Sun, Z., Jiang, Y., Lin, S. (2019): Mitigation of N_2O emissions from urine treated acidic soils by liming. Environmental Pollution, 2019; 255(Pt 1):113237. doi:10.1016/j.envpol.2019.113237.
11. von Uexküll, H.R., Mutert, E. (1995): Global extent, development and economic impact of acid soils. Plant and Soil, 171, 1–15.
12. Hao, T., Q. Zhu, M. Zeng, J. Shen, X. Shi, X. Liu, F.S. Zhang, de Vries, W. (2019): Quantification of the contribution of nitrogen fertilization and crop harvesting to soil acidification in a wheat-maize double cropping system. Plant and Soil, 434, 167 - 184.

13. Zhu, Q., X. Liu, T. Hao, M. Zeng, J. Shen, F. Zhang, de Vries, W. (2020): Cropland acidification increases risk of yield losses and food insecurity in China. Environmental Pollution, (https://www.sciencedirect.com/science/article/pii/S0269749119322560.
14. Woolf, D., Amonette, J., Street-Perrott, A., Lehmann, J., Joseph, S. (2010): Sustainable biochar to mitigate global climate change. Nature Communications, 1, 1-9.
15. Jeffery, S., Verheijen, F.G.A., van der Velde, M., Bastos, A.C. (2011): A quantitative review of the effects of biochar application to soils on crop productivity using meta-analysis. Agriculture, Ecosysystems & Environment, 144, 175-187.
16. Borchard, N., Ladd, B., Eschemann, S., Hegenberg, D., Möseler, B.M., Amelung, W. (2014): Black carbon and soil properties at historical charcoal production sites in Germany. Geoderma, 232–234, 236-242. https://doi.org/10.1016/j.geoderma.2014.05.007.
17. Ye, L., Camps-Arbestain, M., Shen, Q., Lehmann, J., Singh, B., Sabir, M. (2019): Biochar effects on crop yields with and without fertilizer: a meta-analysis of field studies using separate controls. Soil Use and Management, published online doi.org/10.1111/sum.12546.
18. Liu, X., Zhang, A., Ji, C., Joseph, S., Bian, R., Li, L., Pan, G., Paz-Ferreiro, J. (2013): Biochar's effect on crop productivity and the dependence on experimental conditions—a meta-analysis of literature data. Plant and Soil 373, 583–594.
19. Omondi, M. O., Xia, X., Nahayo, A., Liu, X., Korai, P. K., Pan, G. (2016): Quantification of biochar effects on soil hydrological properties using meta-analysis of literature data. Geoderma, 274, 28-34.
20. Major, J., Rondon, M., Molina, D, Riha, S., Lehmann, J. (2010): Maize yield and nutrition during 4 years after biochar application to a Colombian savanna Oxisol. Plant and Soil, 333, 117-128.
21. Kätterer, T., Roobroeck, D., Andrén, O., Kimutai, G., Karltun, E., Kirchmann, H., Nyberg, G., Vanlauwe, B., de Nowina, K.R. (2019): Biochar addition persistently increased soil fertility and yields in maize-soybean rotations over 10 years in sub-humid regions of Kenya. Field Crops Research, 235, pp.18-26.
22. Jeffery, S., Verheijen, F.G., Kammann, C., Abalos, D. (2016): Biochar effects on methane emissions from soils: a meta-analysis. Soil Biology & Biochemistry, 101, pp.251-258.
23. Cayuela, M.L., Van Zwieten, L., Singh, B.P., Jeffery, S., Roig, A. and Sánchez-Monedero, M.A. (2014): Biochar's role in mitigating soil nitrous oxide emissions: A review and meta-analysis. Agriculture, Ecosystems & Environment, 191, pp. 5-16.
24. Fungo, B., Lehmann, J., Kalbitz, K., Thionġo, M., Tenywa, M., Okeyo, I., Neufeldt, H. (2019): Ammonia and nitrous oxide emissions from a field Ultisol amended with tithonia green manure, urea, and biochar. Biology and Fertility of Soils 55, 135–148.
25. Huang, M., Yang, L., Qin, H., Jiang, L., Zou, Y., (2013): Quantifying the effect of biochar amendment on soil quality and crop productivity in Chinese rice paddies. Field Crops Research, 154, 172-177.
26. Nichols, J.D. (1984). Relation of organic carbon to soil properties and climate in the Southern Great Plains. Soil Science Society American Journal, 48, 1382-1384.
27. Six J, Conant R.T., Paul E.A., Paustian, K. (2002): Stabilization mechanisms of soil organic matter: implications for C saturation of soils. Plant & Soil 241, 155–176.
28. Churchman, G.J., Noble, A., Bailey, G., Chittleborough, D., Harper, R., (2014): Clay addition and redistribution to enhance carbon sequestration in soils. In Soil Carbon (pp. 327-335). Springer, Cham.
29. Schiedung, M., Tregurtha, C.S., Beare, M.H., Thomas, S.M., Don, A. (2019): Deep soil flipping increases carbon stocks of New Zealand grasslands. Global Change Biology, 25, 2296– 2309. https://doi.org/10.1111/gcb.14588.

30. Danjuma, M.N., Mohammed, S. (2015): Zai Pits System: A Catalyst for Restoration in the Dry Lands. IOSR Journal of Agriculture and Veterinary Science, 8, 1- 4.
31. Mlih, R., Bol., R., Amelung, W., Brahim, N., (2016): Soil organic matter amendments in date palm groves of the Mena region – a mini review. Journal Arid Land, 8, 77-92.
32. Malézieux, E., Crozat, Y., Dupraz, C, Laurans, M., Makowski, D., Ozier-Lafontaine, H, Rapidel, B., de Tourdonnet, S., Valantin-Morison, M. (2009): Mixing plant species in cropping systems: concepts, tools and models. A review. Agronomy for Sustainainable Development, 29, 43. https://doi.org/10.1051/agro:2007057.
33. Zhou, M., Butterbach-Bahl, K., Vereecken H., Brüggemann, N. (2017): A meta-analysis of soil salinization effects on nitrogen pools, cycles and fluxes in coastal ecosystems. Global Change Biology 23, 1338–1352. doi:10.1111/gcb.13430.
34. Kruse, J., Koch, M., Khoi, C.M., Braun, G., Sebesvari, Z., Amelung, W. (2020): Land use change from permanent rice to alternating rice-shrimp or permanent shrimp in the coastal Mekong Delta, Vietnam: Changes in the nutrient status and binding forms, Science of The Total Environment, 703, art no. 134758; doi.org/10.1016/j.scitotenv.2019.134758.
35. Ferré, M., Müller, A., Leifeld, J., Bader, C., Müller, M., Engel, S., Wichmann, S. (2019): Sustainable management of cultivated peatlands in Switzerland: insights, challenges, and opportunities. Land Use Policy, 87, https://doi.org/10.1016/j.landusepol.2019.05.038.
36. Pan, G., Li, L., Wu, L., Zhang, A. (2004): Storage and sequestration potential of topsoil organic carbon in china\"s paddy soils. Global Change Biology, 10, 79-92.
37. Meersmans, J., Van Wesemael, B., Goidts, E., Van Molle, M., De Baets, S., De Ridder, F., (2011): Spatial analysis of soil organic carbon evolution in Belgian croplands and grasslands, 1960–2006. Global Change Biology, 17, 466-479.
38. Schipper, L.A., Mudge, P.L., Kirschbaum, M.U.F., Hedley, C.B., Golubiewski, N.E., Smaill, S.J., Kelliher,F.M. (2017): A review of soil carbon change in New Zealand's grazed grasslands. New Zealand Journal of Agricultural Research 60, 93-118.
39. McBratney, A., Koppi, T., Field, D.J. (2016): Radical soil management for Australia: a rejuvenation process. Geoderma Regional, 7.
40. Schreiber, C., Schiedung, H., Harrison, L., Briese, C., Ackermann, B., Kant, J., Schrey, S., Hofmann, D., Singh, D., Ebenhöh, O., Amelung, W., Schurr, U., Mettler-Altmann, T., Huber, G., Jablonowski, N.D., Nedbal, L. (2018): On the potential of green alga Chlorella vulgaris to accumulate phosphorus and to fertilize nutrient-poor soil substrates for crop plants. II. Algae cultivation, soil fertilization, and wheat growth. Journal Applied Phycolology, 30, 2827–2836. (doi.10.1007/s10811-018-1390-9).
41. Siebers, N., Hofmann, D., Schiedung, H., Landsrath, A., Ackermann, B., Gao, L., Mojzeš, P., Jablonowski, N.D., Nedbal, L., Amelung, W. (2019): Towards phosphorus recycling for agriculture by algae: Soil incubation and rhizotron studies using 33P-labeled microalgal biomass. Algal Research, 43, art. No. 101634; doi.org/10.1016/j.algal.2019.101634.
42. Crème A., Rumpel C., Malone, S.L., Saby N.P.A., Vaudour, E., Decau, M.L., Chabbi A.: Monitoring grassland management effects on soil organic carbon – a matter of scale. Geoderma, - under review.
43. Angers, D.A., Arrouays, D., Saby, N.P.A., Walter, C. (2011): Estimating and mapping the carbon saturation deficit of French agricultural topsoils. Soil Use and Management 27, 448-452.
44. Wiesmeier, M., Urbanski, L., Hobley, E., Lang, B., Lützow, M., Marin-Spiotta, E., et al., (2019): Soil Organic Carbon Storage as a Key Function of Soils: A Review of Drivers and Indicators at Various Scales. Geoderma, 333, 149-162.
45. Lal, R., Smith, P., Jungkunst H.F., Mitsch, W., Lehmann, J., Ramachandran Nair, P.K., McBratney, A.B., de Moraes Sá J.C., Schneider J., Y. L. Zinn, Skorupa, A.L.A., Zhang, H-L.,

Minasny, B., Srinivasrao, C. & Ravindranath, N.H. (2018): The carbon sequestration potential of terrestrial ecosystems. Journal Soil Water Conservation, 73, 145-152.

46. van Wesemael, B., Paustian, K., Meersmans, J., Goidts, E., Barancikova, G., Easter, M. (2010): Agricultural management explains historic changes in regional soil carbon stocks. Proceedings of the National Academy of Sciences, 107, 14926-14930.
47. Mayer, S., Kölbla, A., Völkelb, J., Kögel-Knabnera, I. (2019a): Organic matter in temperate cultivated floodplain soils: Light fractions highly contribute to subsoil organic carbon. Geoderma 337, 679–690.
48. Steinmann, T., Welp, G., Holbeck, B., Amelung, W. (2016a): Long-term development of organic carbon contents in arable soil of North Rhine–Westphalia, Germany, 1979–2015. European Journal of Soil Science, 67, 616–623. https://doi.org/10.1111/ejss.12376.
49. Steinmann, T., Welp, G. Wolf, A. Holbeck, B., Grose-Ruschkamp, T., Amelung, W. (2016b): Repeated monitoring of organic carbon stocks after eight years reveals carbon losses from intensively managed agricultural soils in Western Germany. Journal Plant Nutrition and Soil Science, 179, 355-366.
50. Crème, A., Rumpel, C., Le Roux, X., Romian, A., Lan, T., Chabbi, A. (2018): Fertilised ley grassland has a legacy effect on soil organic matter quantity and quality and microbial activities favourable to C sequestration. Soil Biology & Biochemistry, 122, 203-210.
51. Kaiser, K., G. Guggenberger. (2000): The role of DOM sorption to mineral surfaces in the preservation of organic matter in soils. Org. Geochemistry, 31, 711-725, doi: 10.1016/S0146-6380(00)00046-2.
52. Cotrufo, M.F., Wallenstein, M.D., Boot, C.M., Denef, K., Paul, E.A. (2013): The Microbial Efficiency-Matrix Stabilization (MEMS)framework integrates plant litter decomposition with soil organic matter stabilization: do labile plant inputs form stable soil organic matter? Global Change Biology, 19, 988–995, doi: 10.1111/gcb.12113.
53. Sokol, N.H., Sanderman, J., Bradford, M.W. (2019): Pathways of mineral-associated soil organic matter formation: Integrating the role of plant carbon source, chemistry, and point of entry. Global Change Biology, 25(1), 12-24, https://doi.org/10.1111/gcb.14482.
54. Six, J., Frey, S.D., Thiet, R.K., Batten, K.M. (2006): Bacterial and fungal contributions to carbon sequestration in agroecosystems. Soil Science Society of America Journal, 70, 555–569.
55. Gulde, S. Chung, H., Amelung, W., Chi, C., Six, J. (2008): Soil carbon saturation controls labile and stable carbon pool dynamics. . Soil Science Society of America Journal, 72, 605–612.
56. Meyer, N., Welp, G., Amelung, W. (2018a): The temperature sensitivity of soil respiration: controlling factors and spatial prediction at regional scale based on environmental soil classes. Global Biogeochemical Cycles, 32, https://doi.org/10.1002/2017GB005644.
57. Kautz, T., Amelung, W., Ewert F., Gaiser, T., Horn, R., Jahn, R., Javaux, M., Kuzyakov, Y., Munch, J. -C, Pätzold, S., Peth, S., Scherer, H.W., Schloter, Schneider, H., Vanderborght, J., Vetterlein, D., Wiesenberg, G., Köpke, U. (2013): Nutrient acquisition from the arable subsoil in temperate climates: a review. Soil Biology & Biochemistry, 57, 1003-1022.
58. Kögel-Knaber, I., Amelung, W., (2014): Dynamics, Chemistry, and Preservation of Organic Matter in Soils. pp. 157-215, In H.D. Holland and K.K. Turekian (eds), Treatise on Geochemistry, Second Edition, vol. 12, Oxford: Elsevier; ISBN 9780080959757. https://doi.org/10.1016/B978-0-08-095975-7.01012-3.
59. Hounkpatin, O., Welp, G., Irénikatché-Akponikpè, P.B., Rosendahl, I., Amelung, W. (2018): Carbon losses from prolonged arable cropping of Plinthosols in Southwest Burkina Faso. Soil & Tillage Research, 175, 51-61.
60. Scharpenseel, H.W., Becker-Heidmann, H.U., Neue, H.U., Tsutsuki, K. (1989): Bomb-carbon, ^{14}C-dating and ^{13}C-measurement s as tracers of organic matter dynamics as of morphogenetic and turbation processes. The Science of the Total Environment, 81/82, 99-110.

61. Paul E.A., Follett, R.F., Leavitt, S.W., Halvorson, A., Peterson, G.A., Lyon, D.J. (1997): Radiocarbon dating for determination of soil organic matter pool sizes and dynamics. Soil Science Society of America Journal, 61, 1058-1067.
62. Rodionov, A., Amelung, W. Peinemann, N., Haumaier, L., Zhang, X., Kleber, M., Glaser, B., Urusevskaya, I., Zech, W. (2010): Black carbon in grassland ecosystems of the world. Global Biogeochemical Cycles, 24: GB3013, doi:10.1029/2009GB003669.
63. Fontaine S., Barot S., Barre P., Bdioui N., Mary B., Rumpel C. (2007): Stability of organic carbon in deep soil layers controlled by fresh carbon supply. Nature, 450, 277-280.
64. Meyer, N., Welp, G. Rodionov, A., Borchard, N., Martius, C., Amelung, W. (2018b): Nitrogen and phosphorus supply controls soil organic carbon mineralization in tropical topsoil and subsoil. Soil Biology & Biochemistry, 119-152-161.
65. Kalbitz, K., Kaiser, K. Fiedler, S. Kölbl, A., Amelung, W., Bräuer, T., Cao, Z.-H., Don, A., Grootes, P., Jahn, R., Schwark, L., Vogelsang, V., Wissing, L., Kögel-Knabner, I. (2013): The carbon count of 2000 years of rice cultivation. Global Change Biology, 19, 1107-1113.
66. Rehbein, K., Sandhage-Hofmann, A., Amelung, W. (2015): Soil carbon accrual in particle-size fractions under Miscanthus x. gigantheus cultivation. Biomass Bioenergy, 78, 80-91.
67. Preger, A., Kösters, R., Du Preez, C.C., Brodowski, S., Amelung, W. (2010): Carbon sequestration in secondary pasture soils: a chronosequence study in the South African Highveld. European Journal of Soil Science, 61, 551-562.
68. Jakobs, I., Schmittmann, O., Schulze Lammers, P. (2017): Short-term effects of in-row subsoiling and simultaneous admixing of organic material on growth of spring barley (*H. vulgare*). Soil Use and Management, 33, 620-630. doi:10.1111/sum.12378.
69. Lal, R. (2003): Soil erosion and the global carbon budget. Environment International, 29: 437-450.
70. Lal, R. (2019): Accelerated soil erosion as a source of atmospheric CO_2. Soil & Tillage Research, 188, 35-40.
71. van Oost, K., Quine, T. A., Govers, G., De Gryze, S., Six, J., Harden, J. W., Ritchie, J. C., McCarty, G. W., Heckrath, G., Kosmas, C., Giraldez, J. V., Marques da Silva, J. R., Merckx, R. (2007): The impact of agricultural soil erosion on the global carbon cycle. Science, 318: 626 - 629.
72. Preston, C.M., Schmidt, M. (2006): Black (pyrogenic) carbon: a synthesis of current knowledge and uncertainties with special consideration of boreal regions. Biogeosciences, 3, 397–420.
73. Marlon, J.R., Bartlein, P.J., Daniau, A.-L., Harrison, S.P., Maezumi, S.Y., Power, M.J., Tinner, W., Vanniére, B. (2013): Global biomass burning: a synthesis and review of Holocene paleofire records and their controls. Quaternary Science Reviews, 65, 5–25.
74. Lehndorff, E., Wolf, M., Litt, T., Brauer, A., Amelung, W. (2015). 15.000 years of black carbon deposition – a post-glacial fire record from lake maar sediments (Germany). Quaternary Science Reviews, 110, 15-22.
75. Batjes, N.H., (1996): Total carbon and nitrogen in the soils of the world. European Journal of Soil Science, 47, 151 – 163.
76. Frolking, S., Roulet, N.T., Tuittila, E., Bubier, J.L., Quillet, A., Talbot, J., Richard, P.J.H., (2010): A new model of Holocene peatland net primary production, decomposition, water balance, and peat accumulation. Earth Syst. Dynam. 1, 1-21.
77. Günther, A., Barthelmes, A., Huth, V., Joosten, H., Jurasinski, G., Koebsch, F., Couwenberg, J. (2020): Prompt rewetting of drained peatlands reduces climate warming despite methane emissions. Nature Communications 11, 1644.
78. Siemens, J., (2003): The European carbon budget: a gap. Science, 502, 1681.

79. Skjemstad, J.O., Spouncer, L.R., Cowie, B., Switf, R.S. (2004): Calibration of the Rothamsted organic carbon turnover model (RothC ver. 26.3), using measurable soil organic carbon pools. Australian Journal of Soil Research 42, 79–88.
80. Zimmermann M, Leifeld J, Schmidt MWI, Smith P, Fuhrer J (2007): Measured soil organic matter fractions can be related to pools in the RothC model. European Journal of Soil Science, 58, 658–667. doi:10.1111/j.1365-2389.2006.00855.x
81. Herbst, M., Welp, G., Macdonald, A. Jate, M., Hädicke, A., Scherer, H.W., Gaiser, T., Herrmann, F., Amelung, W., Vanderborght J. (2018): Correspondence of measured soil carbon fractions and RothC pools for equilibrium and non-equilibrium states. Geoderma, 314: 37-46.
82. Rasmussen, C., Heckman, K., Wieder, W.R., Keiluweit, M., Lawrence, C.R., Berhe, A.A., Blankinship, J.C., Crow, S.E., Druhan, J.L., Pries, C.E.H. and Marin-Spiotta, E. (2018): Beyond clay: towards an improved set of variables for predicting soil organic matter content. Biogeochemistry, 137(3), pp. 297-306.
83. Sachs, J., Remans, R., Smuckler, S., Winowiecki, L., Andelman, S.J., Cassman, K.G., Castle, D., DeFries, R., Denning, G., Fanzo, J., Jackson, L.E., Leemans, R., Lehmann, J., Milder, J.C., Naeem, S., Nziguheba, G., Palm, C.A., Pingali, P.L., Reganold, J.P., Richter, D.D., Scherr, S.J., Sircely, J., Sullivan, C., Tomich T.P. and Sanchez, P.A. (2010): Monitoring the world's agriculture. Nature, 466, 558-560.
84. Baldock, J.A., Hawke, B., Sanderman, J., MacDonald, L.M. (2013): Predicting contents of carbon and its component fractions in Australian soils from diffuse reflectance mid-infrared spectra. Soil Research, 51, 577-595.
85. Rodionov, A., Pätzold, S., Welp, G., Cañada Pallares, R., Damerow, L., Amelung, W. (2014): Sensing of soil organic carbon using visible and near-infrared spectroscopy at variable moisture and surface roughness. Soil Science Society of America Journal, 78, 949-957.
86. Towett, E.K., Shepherd, K.D., Sila, A., Aynekulu, E., Cadisch, G. (2015): Mid-infrared and total X-ray fluorescence spectroscopy complementarity for assessment of soil properties. Soil Science Society of America Journal, 79, 1375-1385.
87. Castaldi, F., Chabrillat, S., Don, A., van Wesemael, B. (2019): Soil organic carbon mapping using LUCAS topsoil database and Sentinel-2 data: An approach to reduce soil moisture and crop residue effects. Remote Sensing, 11 (18), art. no. 2121.
88. Jaconi, A., Poeplau, C., Ramirez-Lopez, L., Van Wesemael, B., Don, A. (2019): Log-ratio transformation is the key to determining soil organic carbon fractions with near-infrared spectroscopy. European Journal of Soil Science, 70, 127 – 139.
