# Supplementary material for: Towards a global-scale soil climate mitigation strategy
Source: Nat Commun. 2020 Oct 27;11:5427. doi: 10.1038/s41467-020-18887-7 (PMC7591914; doi:10.1038/s41467-020-18887-7)
Supplement: Supplementary file 3 — Description of Additional Supplementary Files [file 41467_2020_18887_MOESM3_ESM.pdf]

Title: Supplementary Data 1

Description: Contains 4 excel sheets. Sheet 1 (Metadata) denotes the downloading source addresses for the data used. Sheet 2 (Stations) contains all data used in the Supplementary Figure 1. The data labelled in blue colour refer to the sites exemplarily shown in Table 1, main text. Sheet 3 (Latitude sorted) repeats these data but sorted in the way used to produce Supplementary Figure 1, with values in black assigned to temperate climates, values in blue assigned to subtropical climates, and values in red assigned to tropical climates. Sheet 4 (Correlations (YG vs Cdebt)) shows how yield gap and water limited yield gap (YG) were calculated and extracts the African continent from Supplementary Figure 1.
